# Role of economic evidence in coverage decision-making in South Korea

**DOI:** 10.1371/journal.pone.0206121

**Published:** 2018-10-24

**Authors:** Eun-Young Bae, Hui Jeong Kim, Hye-Jae Lee, Junho Jang, Seung Min Lee, Yunkyung Jung, Nari Yoon, Tae Kyung Kim, Kookhee Kim, Bong-Min Yang

**Affiliations:** 1 School of Pharmacy, Gyeongsang National University, Jinju, Korea; 2 Institute of Pharmacy, Gyeongsang National University, Jinju, Korea; 3 Pharmaceutical Benefit Department, Health Insurance Review and Assessment Service, Wonju, Korea; 4 Health Insurance Policy Research Institute, National Health Insurance Service, Wonju, Korea; 5 Graduate School of Public Health, Seoul National University, Seoul, Korea; Southwest University, CHINA

## Abstract

**Objectives:**

The South Korean government required the submission of economic evidence when it implemented the Positive-List System in December 2006. This study investigates the key factors that influenced actual public insurance reimbursement decisions, including the role of economic evidence, after 10 years of decision practice under compulsory health technology assessment (HTA) for new drugs.

**Method:**

Logistic regression analysis was used to estimate the impact of the variables involved, including cost-effectiveness ratio as a key variable, on reimbursement decisions. The latter were defined as “yes” or “no” at a submitted price and indication. Only cases (n = 91) that present a cost-effectiveness ratio, and that have been reviewed based on this ratio from January 2007 to December 2016, were included in the analysis.

**Results:**

Cases with higher cost-effectiveness ratios were less likely to be accepted. In addition, drugs that were used to treat severe diseases and drugs with no substitute were more likely to be recommended. The probability of acceptance declined along with the level of uncertainty in the submitted evidence. The acceptance rate for severe-disease drugs has increased since 2013, when the government introduced several policies that lowered the existing barriers to positive reimbursement. However, such an increase was not statistically significant.

**Conclusions:**

Cost-effectiveness is one of the most influential factors in drug-reimbursement decisions. However, inclusion of other explanatory variables, in addition to the cost-effectiveness ratio, predicted the results of decisions more accurately.

## Introduction

South Korea was the first Asian country to adopt the cost-effectiveness model when the positive list system came into effect in 2006 [1]. Since Australia first mandated the submission of economic evaluation data for reimbursement decision-making in 1993, many European countries and some Canadian provinces began implementing similar policies [2–4], which later spread to other continents as well [5–8].

The role of economic evaluation differs from country to country. The UK, for example, uses it to identify the most cost-effective use of technology within approved indications. However, Australia, Canada, and other European countries use economic evidence to support their coverage decisions: whether to reimburse or not, and at what price [5, 9]. Some countries require the submission of economic evidence for all drugs, whereas others only require it for drugs that meet the conditions in their country [5, 10].

In South Korea, a cost-effectiveness analysis is required for drugs that are submitted at a higher price compared to their alternatives. For others, the submitted price is compared to that of existing drugs, and accepted only when it is lower than the weighted average of the comparators’ price [1]. Of the 219 cases submitted from January 2007 to December 2011, only 26 were considered based on economic evidence [11]. This number is relatively low compared to other countries that also use economic evidence in reimbursement decisions and pricing [10, 12, 13]. This is because it is not necessary in South Korea to submit economic evidence for the drugs that did not demonstrate superiority in efficacy over their alternatives.

Although there were not many cases in which submissions of economic evaluation data were required, reimbursement decisions on these drugs were sometimes controversial. This is because all of them showed improvements in efficacy or safety compared to existing drugs. Both the patients and clinicians expected these drugs to be listed, and to benefit from them, but not all of them were approved for listing. The main reason for rejection was that the drugs were not cost-effective, which gave the impression that cost-effectiveness ratio was the only determining factor in reimbursement decisions. Is this the case? [14–17]

This study investigates the factors that influence reimbursement decisions, including the role of economic evidence. Only the cases that have presented an incremental cost-effectiveness ratio (ICER), and that have been reviewed based on this ratio, are included in our analysis.

Previously, Bae et al. provided a descriptive analysis of the final reimbursement decisions from January 2007 to December 2014, as well as their comparative effectiveness, but did not analyze the impact of ICER and other factors [1]. Park et al. analyzed the submissions that were reviewed for the first 2 years of the positive list system (PLS), but did not include ICER as an independent variable [18].

This study analyzes the impact of ICER in South Korea and complements Park’s study by including the most recent submissions to the HIRA.

## Materials and methods

### Data

The Korean drug reimbursement decision-making process is comprised of two stages. If a company submits the dossiers for coverage, the Health Insurance Review and Assessment service (HIRA) reviews them, and makes a coverage decision based on the Pharmaceutical Benefit Coverage Advisory Committee’s (PBCAC) recommendation. The PBCAC, independent advisory committee, considers the comparative effectiveness and cost-effectiveness of the drugs submitted for review and recommends the listings to HIRA. In case the submission includes the cost-effectiveness analysis, the Economic Sub-Committee (ESC) assesses the technical aspects of the submitted studies before the PBCAC meeting.

Accepted cases are finally listed only after price negotiations between the submitting company and the National Health Insurance Service (NHIS) have been successful.

Our analysis included all the cases that submitted complete economic evaluation data from January 2007 to December 2016 and for which coverage decisions were made by HIRA before price negotiation based on the data submitted. The authors were permitted to access data only for research that was aimed at evaluating the PLS.

Data were extracted using a pre-determined format. Abstracted information included the brand and generic names of the drugs, the dates of the meetings, the history of prior decisions, the reason for recommendation, the severity of the disease, availability of alternative treatments, ICER, and budget impact. Among these, some data, such as ICER and budget impact, are regarded as confidential by HIRA. Accordingly, the co-authors affiliated with HIRA extracted data from the minutes of the PBCAC and the documents prepared for the ESC meeting. To ensure the accuracy of the data extraction, the two authors independently extracted data according to a predefined extraction form. If their judgments did not agree with each other, a final conclusion was drawn through mutual discussion.

In cases where there were multiple numbers available for ICER and budget impact, the authors met with responsible HIRA staff to discuss and clarify which ones were actually considered by PBCAC.

### Variables

In this study, reimbursement decisions are defined as “yes” or “no” for the submitted price and indication. In cases where the drug was recommended to be listed, but with a condition requiring the lowering of the price, the decision was defined as “no” because the submitted cost-effectiveness ratio was not acceptable at that price.

When the same drug was submitted and evaluated several times, each case was included in the analysis separately, as each submission included different ICER, and the decisions were made based on different information provided. In cases where drugs with multiple indications were submitted, each indication was also treated separately, applying the same logic. However, these related submissions have commonalities in many aspects, and the reimbursement decisions that were made may not have been independent of each other. Therefore, robust standard errors were calculated using correlations among the Pearson residuals within drugs and used in the analysis to account for this clustering effect.

If a drug had been submitted before, there was a possibility that the latest decision could be influenced by previous decisions, increasing the likelihood of acceptance. This is because it had already been considered by the committee, and the sponsoring companies already knew the critical issues when they decided to resubmit their applications. They would either lower the price, or submit supplementary evidence to support their claims to increase the likelihood of acceptance in the PBCAC’s consideration. Therefore, our analysis included a variable that identifies whether the case was first submitted.

In South Korea, cost per quality-adjusted life years (QALYs) gained is the preferred measure used to represent the cost-effectiveness of a drug. Among the reviewed cases, however, 10 have presented just cost per life years gained. In the analysis, both measures were included without any adjustments, as the decision-makers considered only the numbers presented and it was difficult to determine the relativeness between cost per life years gained and cost per QALYs gained [19].

ICER was provided by the manufacturers. If the reviewers raised questions about the validity of the ratio, and suggested an alternative number instead of a submitted one, the reviewers’ choice was cited. If the ratio was questioned but no alternative number was provided, the number originally submitted by the manufacturer was used.

In addition to cost-effectiveness, multiple factors are known to have been considered in reimbursement decision-making. In an interview with HIRA’s senior reviewers, the following five factors were suggested as the most influential criteria in drug reimbursement decision-making: clinical improvement, cost-effectiveness, the severity and rarity of disease, and the availability of substitutes. This was consistent with the findings in a survey conducted in a previous study [1, 11]. Keeping these factors in mind, the following variables have been included in our analysis. This list of variables was developed with reference to previous studies evaluating the performance of PLS in Korea [1, 11, 18] as well as studies investigating factors contributing to reimbursement decisions in other countries [19–22] and the factors identified by the HIRA reviewers (Table 1).

**Table 1:** The definition of variables.

Variables	Coding	Definitions
Decisions	1 = Accepted 0 = Rejected	Recommendations made by PBCAC; Accept or reject cases submitted for listing.
ICER	Numeric: KRW million/QALY gained	The incremental cost-effectiveness ratio that was submitted by pharmaceutical companies and considered by PBCAC as the number that reflects the submitted drug’s cost-effectiveness. In case only data for cost per life years gained was available, it was alternatively considered.
Prior Submissions	1 = Considered before 0 = First submitted	This variable represents the submission history; if the case was previously considered by PBCAC.
Severity of Diseases	1 = Diseases that are included in severe disease category 0 = Others	The severity of disease was defined as a dichotomous variable. The severe diseases were defined as cancer, rare diseases, or a disease in a terminal stage. If the diseases were not included in the category of severe disease, they were categorized as others.
Availability of Substitutes	2 = No substitutes 1 = Limited substitutes 0 = Others	If the alternative drugs were available for the same indication. If there were substitutes available, but the substitutability is limited for the difference of effectiveness or side effects, the cases were defined to have limited substitutes.
Budget Impact	Numeric: KRW billion	Budget impact was measured as anticipated increases in drug expenditure three years after listing.
Uncertainty	2 = Very uncertain 1 = Uncertain 0 = Others	If the committee judged the number was too uncertain to make a decision based on it, it was categorized as ‘very uncertain’; ‘uncertain’ if the committee raised uncertainty issues in its deliberation, but based its decision depending on the submitted number.
Availability of head-to-head trials	1 = Yes 0 = No	If there were head-to-head trials with comparators. This was used to identify If the main efficacy was supported by direct evidence
Timing of Submission	1 = After Dec. 2013 0 = Before Dec. 2013	If the submission was deliberated before Dec.2013 or after Dec.2013.

PBCAC: Pharmaceutical Benefit Coverage Assessment Committee; ICER: Incremental Cost-Effectiveness Ratio; QALY: Quality-Adjusted Life Years. 1,000 KRW = 0.88 US$

#### Severity of disease

The severity of the disease is the most widely used priority setting criterion in health care. However, there is no simple index to measure it, and previous studies have also provided different definitions of severity [20–22]. Therefore, we classified the severity level to align with the government’s policies. The Korean government announced that the National Health Insurance (NHI) would cover the costs of all treatments required for several very serious diseases, and will improve access to new drugs for cancer or other rare diseases through a risk-sharing scheme. Meanwhile, in PBCAC’s consideration, the severity level was judged based on the remaining life expectancy with current treatment. If this number is less than 2 years, it is assumed to be very severe. Therefore, if the disease falls under categories like cancer or rare diseases, or the remaining life expectancy is less than 2 years, it is classified as “severe,” and all others as “not severe.”

#### Budget impact

According to the guidelines, the budget impact, measured as expected drug expenditure three years after listing, is among the factors that are expected to be considered in PBCAC’s deliberation.

#### Availability of substitutes

The availability of substitutes reflects the extent of unmet needs. If there is no alternative treatment available, the unmet needs for the disease was considered to be high, and was more likely to be accepted in reimbursement decision-making. Drugs that have a few substitutes available could still limit the scope of substitution because there is a large efficacy gap between the existing drugs and the new drugs or for some patients; the drug may not be substitutable. Thus, the drugs were classified into three categories, according to the availability of substitutes: 0 for drugs that have enough substitutes, and 1 for drugs that have substitutes limited in substitutability, and 2 for drugs that do not have any alternative treatments.

#### Uncertainty

There are various ways of including “uncertainty” in the model. Dakin et al., for example, used the ICER range—the difference between minimum and maximum ICER [20]—while Devlin et al. used the number that was calculated by dividing the range of ICER by the base case ICER [21], and Harris et al. used the upper limit in a sensitivity analysis [22]. We used the committee’s judgment on the uncertainty of ICER instead of using the range or upper value of it. This was because the upper value is correlated with the base case ICER, and if the value were lower than the threshold, it would be accepted even if the range was high. The overall evaluation of the committee is rather subjective, but it reflects the committee’s judgment on uncertainty more directly compared to a range or the other criteria used in previous studies.

In the analysis, the cases were categorized as “very uncertain” if the committee judged the ICER to be too uncertain to make a decision based on it, and “uncertain” if the committee raised issues of uncertainty in a committee meeting. In addition, as an indirect way of reflecting uncertainty, the availability of head–to-head evidence on efficacy is also included in our analysis.

#### Timing of submission (policy change)

In 2013, the Ministry of Health and Welfare (MOHW) announced several policy changes, which included the introduction of a risk-sharing scheme and an increase in the upper ICER threshold. These policies mainly target cancer and rare diseases. The policy change was expected to influence the positive recommendation rate of the committee, so the variable that divided the periods into “before” and “after” 2013 is included in the model.

### Analysis

Logistic regression analysis was used to estimate the probability of acceptance for a unit change of each explanatory variable. The relationship between the probability of acceptance and a set of explanatory variables is as follows:

$$P(y_{i}\;=\;1|x_{i})\;=\;\frac{exp\;(x_{i}\beta )}{1+exp\;(x_{i}\beta )}$$

(where y_i_ is coded 1 for accepted cases, and 0 for others).

In model 1, only ICER is included as an explanatory variable. Meanwhile, model 2 is constructed with all independent variables identified as influential in previous studies. Model 3 added likely interaction to model 2. ICER is always included in our analysis, as it is the key factor that our analysis is exploring for its impact.

The performance of the model is evaluated with the proportion of cases that were correctly classified, comparing predictions to real decisions [20]. For this, the probability of acceptance for each submitted case was estimated, and was compared to the real decisions. If the estimated probability is higher than 0.5, and the case was accepted in the real decision-making process, the model is regarded as predicting the result correctly, and if not, vice versa.

The same data set was used to test model performance, as there was not sufficient data for a separate test. Pseudo-R2 and AIC were also presented. In all model specifications, robust standard error was estimated considering the probable clustering of decisions [20–22].

## Empirical results

### Univariate analysis

Table 2 shows the distribution of decisions by category for each variable. The cases with a history of prior submission tended to have a slightly higher acceptance rate than the ones that were submitted for the first time, but the difference was not significant.

**Table 2:** Univariate association between PBCAC decisions and predictors.

Variables	PBCAC’s decision		Total n (%)	p-value
	Accept n (%)	Reject n (%)		
ICER (KRW million)				
Mean (SD)	23.1 (16.2)	58.3 (53.8)	40.5 (43.1)	<0.000
• Less than 25	29 (70.7)	12 (29.3)	41 (100.0)	<0.000
• 25 to 50	17 (54.8)	14 (45.2)	31 (100.0)	
• More than 50	0 (0.0)	19 (100.0)	19 (100.0)	
Prior submission				
• First submission	28 (46.7)	32 (53.3)	60 (100.0)	0.303
• Considered before	18 (58.1)	13 (41.9)	31 (100.0)	
Severity of disease				
• Severe	21 (43.8)	27 (56.3)	48 (100.0)	0.170
• Not severe	25 (58.1)	18 (41.9)	43 (100.0)	
Availability of substitutes				
• No substitute	7 (58.3)	5 (41.7)	12 (100.0)	0.845
• Limited substitute	27 (49.1)	28 (50.9)	55 (100.0)	
• Others	12 (50.0)	12 (50.0)	24 (100.0)	
Budget Impact (KRW billion)				
Mean (SD)	9.6 (17.8)	13.0 (18.7)	11.3 (18.2)	0.380
• Less than 1	6 (46.2)	7 (56.8)	13 (100.0)	0.085
• 1 to 5	23 (67.6)	11 (32.4)	34 (100.0)	
• 5 to 10	7 (36.8)	12 (63.2)	19 (100.0)	
• More than 10	10 (40.0)	15 (60.0)	25 (100.0)	
Uncertainty				
• Very uncertain	3 (10.7)	25 (89.3)	28 (100.0)	<0.000
• Uncertain	25 (62.5)	15 (37.5)	40 (100.0)	
• Others	18 (78.3)	5 (21.7)	23 (100.0)	
Availability of head-to-head trials				
• Yes	37 (50.7)	36 (49.3)	73 (100.0)	0.958
• No	9 (50.0)	9 (50.0)	18 (100.0)	
Timing of submission				
• After Dec. 2013	25 (50.0)	25 (50.0)	50 (100.0)	0.908
• Before Dec. 2013	21 (51.2)	20 (48.8)	41 (100.0)	

PBCAC: Pharmaceutical Benefit Coverage Assessment Committee; ICER: Incremental Cost-Effectiveness Ratio; SD: standard deviation. Numbers in parenthesis are row percentages. 1,000 KRW = 0.88 US$.

By the end of 2016, a total of 91 cases were reviewed by the ESC, of which 46 were accepted, and 45 were rejected. In the accepted cases, the average ICER was KRW 23.1 million (1,000 KRW = 0.88 USD), which was lower than that of the rejected cases. Although the explicit ICER threshold is not published in Korea, it is known that the per capita GDP level is considered in the deliberation process. In other words, if the additional cost per QALY gained exceeds the per capita GDP level (KRW 25 million), it is unlikely to be considered cost-effective. However, some cases, wherein ICER exceeded per capita GDP, were still accepted. Of the 46 accepted cases (in total), 17 (40.0%) were in this case, but none of them had an ICER that was more than double the per capita GDP. Meanwhile, 12 cases were rejected for uncertainty, even though their ICER was lower than the per capita GDP. Nineteen cases had an ICER that was more than double the per capita GDP, but all of them were rejected and subsequently not listed.

Forty-eight out of the 91 cases had indications of cancer or other severe diseases, and 12 cases did not have any substitutes available. In univariate analysis, the drugs used for severe diseases were rejected at a higher rate than those for non-severe diseases, while drugs with no alternatives available were rejected at a lower rate than others. However, both differences were not significant at α = 0.05.

Average financial cost to NHI was KRW 11.3 billion, but there was no significant difference between accepted and rejected cases.

When the uncertainty of ICER was judged depending on the committee’s overall evaluation, 28 were grouped as “very uncertain,” and 40 as “uncertain,” and all showed a significant association with decisions. While judging uncertainty was based on the availability of evidence, there was no significant difference in the acceptance rate between the two categories. Timing of the submission also did not result in any difference in acceptability between the groups. However, the mean ICER of cases accepted before 2013 was KRW 13.5 million, and KRW 34.5 million for cases accepted after 2013.

### Logistic regression

Table 3 displays the odds ratio and confidence intervals of the included variables in the multivariate logistic regression model (model 1–3). Model 1 included only ICER, and model 2 included ICER and other variables known to affect reimbursement decision-making in previous studies. These other variables included prior submission, severity of disease, the availability of substitutes, budget impact, uncertainty, the availability of head-to-head trials, and the timing of submission. Model 3 added a more likely interaction term to model 2.

**Table 3:** Multivariate logistic regression analyses of PBCAC decisions.

Variables	Model 1	Model 2	Model 3
	OR(95% CI)	OR(95% CI)	OR(95% CI)
ICER	0.967(0.952–0.982)***	0.878(0.810–0.951)***	0.855(0.753–0.971)**
Prior submission		1.393(0.119–16.266)	1.235(0.11–13.390)
Severity of disease		19.110(0.238–999.999)*	11.805(0.814–168.209)*
Availability of substitutes			
• No substitute		7.936(0.953–66.072)*	25.552(1.439–453.591) **
• Limited substitute		1.872(0.150–23.332)	1.996(0.193–20.636)
Budget impact		1.012(0.969–1.058)	1.027(0.969–1.089)
Uncertainty			
• Very uncertain		0.003(0.000–0.085)***	0.002(0.000–0.232)**
• Uncertain		0.672(0.141–3.197)	0.921(0.226–3.753)
Availability of head-to-head trials		0.304(0.040–2.285)	0.306(0.051–1.827)
Timing of submission		1.562(0.087–28.186)	0.411(0.013–12.925)
Severity of disease*No substitute			0.015(0.000–0.502)**
Severity of disease*Limited substitute			0.485(0.036–6.534)
Severity of disease*Timing of submission			68.607(0.270–21155.094)
N	91	91	91
Log likelihood	20.0036***	75.8935***	82.8911***
Pseudo R^2^ AIC	0.159 110.138	0.602 71.251	0.657 72.248
Sensitivity	76.1	91.3	91.3
Specificity	57.8	80.0	86.7
% Correctly classified	67.0	85.7	89.0

PBCAC: Pharmaceutical Benefit Coverage Assessment Committee; OR: Odds ratio, CI: Confidence interval, ICER: Incremental Cost-Effectiveness Ratio. Standard errors were calculated using robust method.

* p<0.10.

** P<0.05.

*** p<0.001.

In model 1, ICER had a significant impact on the committee’s decisions, and it alone correctly predicted the direction of recommendation in 67.0% of cases.

Model 2 showed a significant improvement in predictability compared to model 1. 85.7% of cases were correctly classified in Model 2. ICER, the availability of substitutes, and uncertainty had a significant impact on the committee’s decision at the significance level of 0.1. Other factors did not have a significant impact contrary to our expectations, but the direction of impact was as expected.

Model 3, which included the interaction term of severity with the availability of substitutes and timing of submission, did not show a significant improvement in predictability compared to model 2 (85.7% vs. 89.0%).

**Fig 1:**
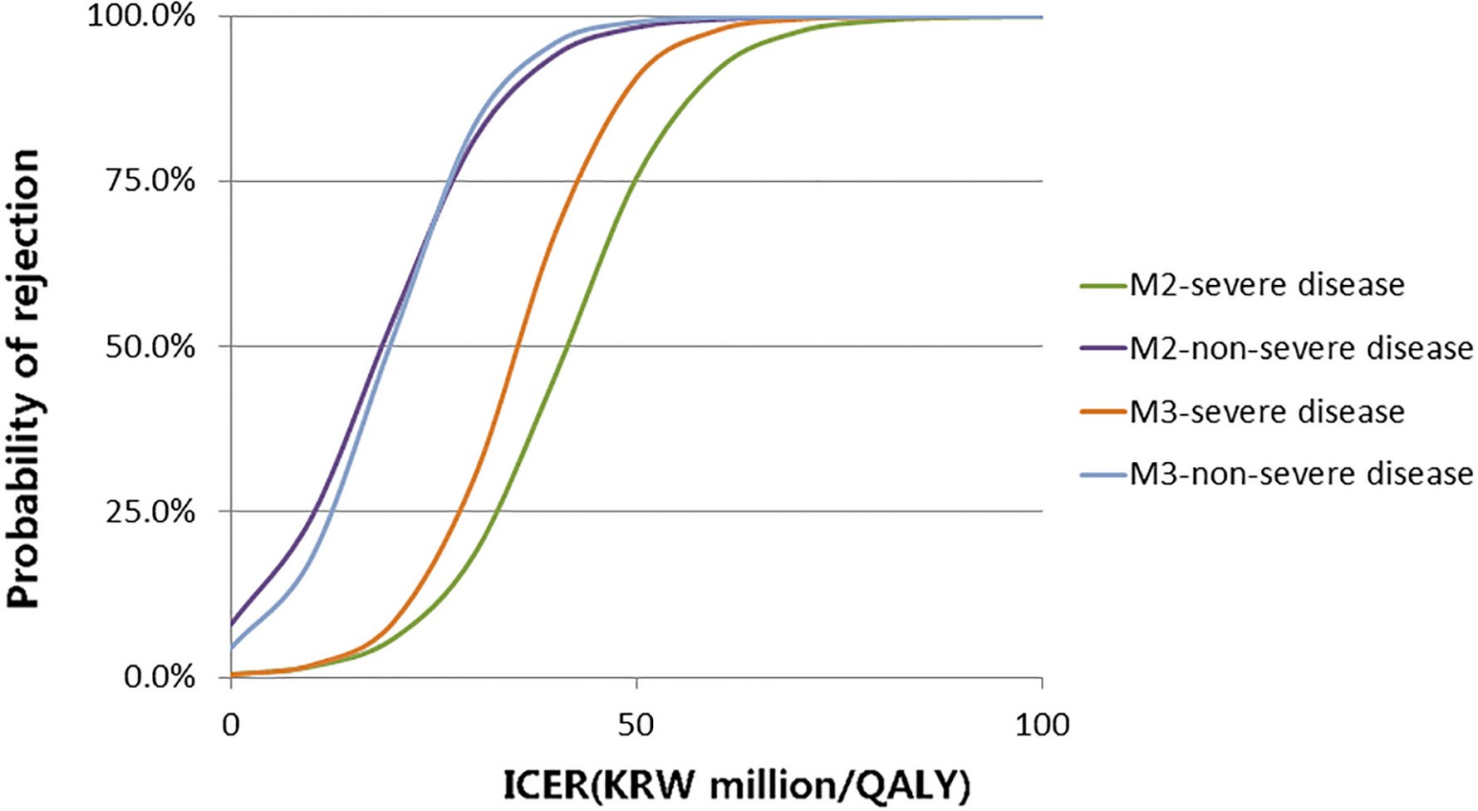
Predicted probability of rejection by cost per quality-adjusted life years.

In model 3, more variables were significant than in model 2. The severity of disease and interaction term of severity with availability of substitutes had a significant impact on the decision in model 3. However, the coefficient of the interaction term of severity and the availability of substitutes were very different from the expected values and, thus, it is difficult to interpret the results, which may have been caused by the small sample size. Interaction terms of severity with timing of submission included in model 3 were not significant. It can be inferred from the coefficient, then, that the probability of acceptance for severe disease drugs has increased after 2013;

ICER is included in all models, and clearly had a significant effect on all models. When other variables are controlled, increasing ICER by KRW 1 million decreased the probability of acceptance by 12% in model 2, and 14% in model 3. Fig 1 shows the change in probability of rejection according to the changes in ICER with other variables controlled at the baselines in model 2 and model 3, where the continuous variable had an average value and the dummy variables had a value of 0 except for availability of head-to-head trials. ICER, which has a 50% chance of getting a positive recommendation on the curve, amounts to KRW 42.3 million per QALY for drugs used to treat severe diseases, while KRW 19.7 million per QALY for non-severe diseases in model 2. Model 3 has values of KRW 37.2 million per QALY and KRW 21.5 million per QALY respectively.

Besides ICER, other factors also affected the decisions. In model 2, for example, the drugs used for severe diseases were 19 times more likely to be accepted by the committee compared to those for non-severe diseases, and drugs with no substitute were 8 times more likely to be recommended by the committee compared to drugs with substitutes. Cases judged to be “very uncertain” by the committee were less likely to be accepted by 0.3% of others. On the other hand, cases where uncertainty was raised but not judged to be “very uncertain” were not different from others.

## Discussion

Our analysis found that the cost-effectiveness ratio was the most influential factor, but that severity of disease, availability of substitues and uncertainty of submitted evidence were also considered in PBCAC’s reimbursement decision-making. Drugs used to treat severe diseases were more likely to be accepted by PBCAC, and drugs with very weak evidence on ICER were less likely to be accepted when other variables were controlled.

However, contrary to our expectations, some variables did not show a significant impact on decisions. For instance, budget impact was not significant, even though it is one of the official decision making criteria. For this result, HIRA staff mentioned that budget impacts were not considered important in PBCAC’s decisions if they were not significant, as they were mainly used in price negotiations between the company and NHIS [11].

In this paper, we are particularly interested in the impacts of changes in government policies on individual reimbursement decisions. In 2013, the South Korean government decided to implement risk-sharing arrangements and to heighten the maximum allowable ICER level as measures of improving the coverage of NHI for several severe diseases.

Risk-sharing schemes were introduced to allow the submission to meet the cost-effectiveness criteria by lowering the effective prices. These policies have been implemented in many countries to enhance the accessibility to high-priced drugs. Application can be made for a life-threatening anti-cancer drug, or orphan drug without any substitutable alternatives, to be part of the risk-sharing plan. Until September 2017, 31 drugs (21 molecules) have been listed in accordance with the risk-sharing plan [23].

In addition to risk sharing, the increase in the upper ICER threshold provided more flexibility in PBCAC’s deliberation, and thereby other social values could be considered more important than before.

The risk-sharing scheme and the ICER threshold adjustment were generally considered to have improved access to high-priced drugs compared to the previous period—especially anti-cancer drugs. The odds ratio of the timing of the submission with severity of disease in this study supported this assumption; the likelihood of drugs used for severe diseases to be listed had increased after 2013 when the two policies were introduced, even though both coefficients were not significant.

In addition to exploring the factors affecting coverage decisions, the probability-based cost per QALY threshold was also calculated in this study. This does not coincide with the median or maximum ICER of accepted cases, as other factors were controlled in the analysis; the ICER was estimated at fifty-fifty for acceptance or rejection.

As expected, the 50% acceptance-ICER is higher in the severe disease group compared to the non-severe disease group. Model 2 and model 3 showed a similar trend, but the number in model 2 is somewhat different from that in model 3 for the severe disease group. This may be because the variation increases for these drugs. For some submissions, the ICER was higher than KRW 100 million per QALY, and most of them were for severe diseases.

Although finding an explicit threshold is not the aim of this study, the results can be used to generate implications when specific cases are deliberated.

This study is limited in some aspects. First, there are some variables for which it is difficult to establish a clear criterion for defining their level. Uncertainty, availability of substitutes, and severity of disease all correspond to such variables.

For uncertainty, we made a decision based on the committee’s judgement, however, it is difficult to judge the degree of uncertainty with wording alone, because different reviewers wrote evaluation reports at different times. This was inevitably subject to subjective judgment in data extraction.

For severity of disease, we made them dichotomous to classify the degree of each variable more explicitly, but this made it difficult to capture the impact of subtle changes in each variable.

In addition, there may be other unknown factors that could influence decision-making. However, this study did not address all of these factors.

## Conclusions

Cost-effectiveness has emerged as the most important factor in reimbursement decision-making of drugs in South Korea. This study confirmed this through empirical data. In addition, this study found that the severity of disease, the availability of substitutes, and the uncertainty of evidence also had an impact on the committee’s decisions, and the impact of the severity of disease was greater after 2013 when the government implemented new policies, such as the introduction of risk-sharing schemes and an increase in the upper ICER threshold. However, there were not many significant variables, and the results of analysis showed substantial variations according to the model adopted, which may be as a result of the small sample size. The small sample size issue can be overcome if more data are accumulated in the future, and it is expected that more stable results will be obtained through further analysis.

## Supporting information

S1 Table List of cases reviewed by Pharmaceutical Benefit Coverage Assessment Committee. (XLSX)
